# DJ-1 as a Modulator of Autophagy: An Hypothesis

**DOI:** 10.1100/tsw.2010.165

**Published:** 2010-08-17

**Authors:** Rosa A. González-Polo, Mireia Niso-Santano, Ruben Gómez-Sánchez, Jose M. Bravo-San Pedro, José M. Fuentes

**Affiliations:** Centro de Investigacion Biomedica en Red Sobre Enfermedades Neurodegenerativas (CIBERNED), Departamento de Bioquímica y Biología Molecular y Genética, E.U. Enfermería y T.O., Universidad de Extremadura, Cáceres, Spain

**Keywords:** DJ-1, autophagy, neurodegeneration, Parkinson’s disease

## Abstract

The etiology of Parkinson's disease (PD) is not completely defined, although environmental factors (for example, exposure to the herbicide paraquat [PQ]) and genetic susceptibility (such as DJ-1 mutations that have been associated with an autosomal-recessive form of early-onset PD) have been demonstrated to contribute. Alterations in macroautophagy have been described in the pathogenesis of this neurodegenerative disease. We have established a model system to study the involvement of the DJ-1 protein in PQ-induced autophagy. When we transfected cells exposed to PQ with DJ-1–specific siRNA, we observed an inhibition of the autophagic events induced by the herbicide, as well as sensitization additive with PQ-induced apoptotic cell death and exacerbation of this cell death in the presence of the autophagy inhibitor 3-methyladenine. These results suggest, for the first time, an active role for DJ-1 in the autophagic response produced by PQ, opening the door to new strategies for PD therapy.

DJ-1 (CAP1/RS/PARK7) is a molecule that occupies a pivotal position in cellular biology. A loss or gain of its function drives abnormal cellular responses, leading either to cell death in neurodegenerative disease or to unregulated cell survival in cancer[1]. Deletions and loss-of-function mutations in the human DJ-1 gene have been associated with autosomal-recessive early-onset Parkinson's disease (PD)[2,3,4]. Moreover, DJ-1 immunoreactive inclusions are found in other α-synucleinopathies and tauopathies, indicating that different neurodegenerative diseases might share a common mechanism in which DJ-1 might play a key role[5]. While the pathogenesis of PD and other neurodegenerative diseases is still unknown, the combination of an environmental insult (for example, paraquat [PQ] exposure[6]) and genetic susceptibility is suspected to play an important role. Moreover, ultrastructural examination has revealed that characteristics of apoptosis and autophagic degeneration are present in melanized neurons of the substantia nigra in PD patients[7]. Previous studies by our group have established a possible link between oxidative damage, autophagy, and apoptotic death using human neuroblastoma SH-SY5Y cells treated with PQ[8,9]. In this system, we have shown that low concentrations of PQ produce a reactive oxygen species (ROS) burst, followed by the biochemical and morphological characteristics of

*These authors contributed equally to this paper.
**Corresponding author.
©2010 with author.
Published by TheScientificWorld; www.thescientificworld.com autophagy. However, although the pesticide initially induced the morphological appearance of autophagy, the cells subsequently displayed morphological and biochemical changes that are typical of apoptotic cell death, such as phosphatidyl-serine exposure on the outward-facing side of the plasma membrane, decrease of the mitochondrial membrane potential, release of cytochrome c from the mitochondria to cytosol, caspase 3 activation, or nuclear condensation.

The purpose of the recent studies by our group was to investigate the possible involvement of the DJ-1 protein in this PQ model system, studying a possible interaction between the herbicide PQ and a gene related to PD, namely DJ-1. There are several studies that support the idea that DJ-1 promotes cell survival by protecting cells from oxidative stress. DJ-1 overexpression confers resistance to oxidative stress, while a decrease in the level of DJ-1, due either to RNA interference (RNAi)[10] or knockout of the DJ-1 gene, enhances cytotoxicity mediated by hydrogen peroxide ($H_2O_2$) and MPTP (1-methyl-4-phenyl-1,2,3,6-tetrahydropyridine)[2]. DJ-1 is thus considered to be an antioxidant protein, consistent with its ability to quench ROS and to acquire a more acidic isoelectric point following oxidative stress[11]. Our recent observations[12] are in agreement with these studies. We have seen that transfection with DJ-1–specific siRNA causes loss of viability in neuroblastoma cells and that this effect is accelerated in the presence of PQ. Moreover, PQ produces an important dose-dependent increase of the DJ-1 protein levels, correlated with mitochondrial cytochrome c release and chromatin condensation. This finding highlights the importance of DJ-1 in conferring a neuroprotective effect against PQ-induced toxicity and supports the studies that suggest that the loss of DJ-1 protein activity makes neurons more vulnerable to oxidative stress generated by PQ, a well-known inducer of such oxidative stress[13,14]. Moreover, it has been reported that a specific cleavage of DJ-1 occurs under oxidative conditions and this could explain the effect seen in the cells treated with PQ in our system[15]. The physiological significance of cleaved forms of the *Xenopus* homolog and human DJ-1 remains unclear. It would be interesting to examine the role of cleaved DJ-1 in terms of defense reaction against various stresses.

PQ induces molecular events compatible with autophagy. There are studies that demonstrate that macroautophagy, an evolutionarily conserved pathway of lysosomal degradation of damaged proteins and organelles[16,17], plays a significant role in the MPP+ model of parkinsonian injury[18,19]. These data are very interesting due to the similarity of the chemical structures of MPP+ and PQ. While neurons undergo degeneration when basal autophagic degradation is disrupted[20,21], increased autophagy can also contribute actively to neurodegeneration[22,23,24]. On the other hand, there is evidence linking the role of genes that encode proteins involved in inherited forms of PD to a protein degradation mechanism, such as the autophagic response[25,26,27]. Studies by Plowey and colleagues[28] have demonstrated an active role for autophagy in neurite outgrowth associated with the expression of mutant G2019S-LRRK2 (this leucine-rich repeat kinase 2 mutation is implicated in autosomal-dominant and some cases of sporadic PD) in neuroblastoma cells differentiated with retinoic acid. Given that there was already evidence that DJ-1 is involved in the regulation of proteasome function, and therefore protein clearance[29,30], we wondered whether DJ-1 might be implicated in the autophagic process. We found that DJ-1 RNA interference knockdown not only abolished the cytoplasmic accumulation of PQ-induced autophagic vacuoles, but also inhibited accumulation of the autophagosome marker LC3-II and increased the activation of phosphorylated mTOR and its substrate P-p70$^{S6K}$ (Fig. 1). These results suggest an important role for DJ-1 in PQ-induced autophagy. There are not many studies relating DJ-1 protein directly with autophagy[31]. Cookson and coworkers postulated that the RNA binding activity of DJ-1 supports its involvement in multiple cellular pathways, suggesting that DJ-1 may play a role in coordinating responses to oxidative damage and suppression of cell death[32].

Recently, Vasseur and colleagues[1] demonstrated that wild-type (WT) levels of DJ-1 expression are required for proper turnover of p62, a marker of autophagy, which is selectively incorporated into autophagosomes and is efficiently degraded by autophagy induced by hypoxia[33]. Without DJ-1, autophagy in response to lowered oxygen availability is partly impaired as it is not accompanied by p62 down-regulation. In Vasseurs' study, the lack of suitable LC3 processing and p62 clearance in DJ-1 knockdown cells suggested a neuroprotective role for DJ-1 in hypoxic autophagy. Moreover, in our study, we observed that 3-methyladenine (3-MA) accelerated the cell death induced by PQ and DJ-1 siRNA,

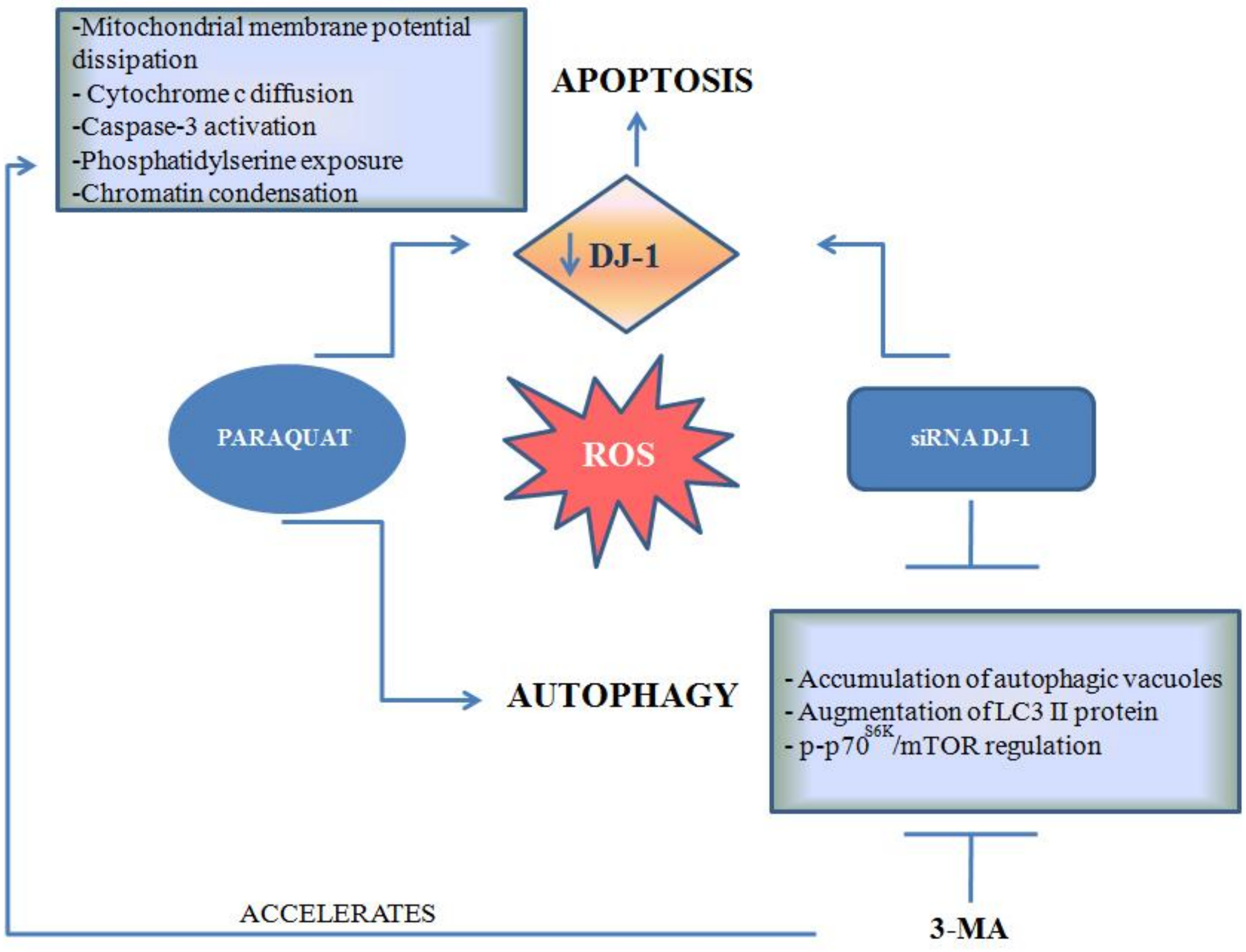

**FIGURE 1**. Schematic of the connections between the DJ-1 protein and other factors in the PQ neurotoxicity model. The toxicity mediated by PQ is produced via ROS. In the first instance, the cell could activate an autophagic defense mechanism, including the accumulation of autophagic vacuoles and augmentation of LC3 II protein regulated via p-p70$^{S6K}$/mTOR. Later, however, coinciding with a decline in DJ-1 levels, the cells succumb to apoptotic death with mitochondrial membrane potential dissipation, cytochrome c diffusion, caspase-3 activation, phosphatidylserine exposure, and chromatin condensation. DJ-1 siRNA accelerated the apoptotic cell death induced by PQ to the extent that the autophagic defense response was inhibited. Autophagy inhibition with 3-MA accelerated the apoptotic cell death induced by the combination of stimuli.

suggesting that autophagy plays a neuroprotective role. This observation, already described by us[34,35] and by other authors[27], allows one to postulate that stimulating autophagy may constitute a new therapeutic strategy for the treatment of PD.

In conclusion, we have established a model system to study the involvement of the DJ-1 protein in PQ-induced autophagy[12]. When cells transfected with DJ-1–specific siRNA were treated with PQ, we observed an inhibition of the autophagic events otherwise induced by the herbicide, as well as sensitization additive with PQ-induced apoptotic cell death and exacerbation of this apoptotic cell death in the presence of the autophagy inhibitor 3-MA (Fig. 1). These results suggest, for the first time, that DJ-1 stimulates the autophagic response to PQ, reduces the apoptotic cell death response to PQ at the same time, and, therefore, that autophagy induced by DJ-1 may be protective vs. apoptotic. These observations open the door to new strategies of investigation for therapy of PD and of other pathologic processes in which factors that induce apoptosis are prevalent.

## ACKNOWLEDGMENTS

This work was supported by the grants PI070400 and CP08/0010 (FIS, Ministerio de Ciencia e Innovación, Spain). R.A.G-P. was supported by a “Miguel Servet” contract. M.N-S. was supported by a CIBERNED predoctoral contract. J.M.B-S was supported by Junta of Extremadura predoctoral fellowship. R.G.-S. was supported by FPU predoctoral fellowship Ministerio de Educación, Spain. The authors would like to thank P. Delgado and R. Ronco for invaluable technical assistance. The authors also thank FUNDESALUD for helpful assistance.
